# Genetic Variation and Evolutionary Analysis of Eggplant Mottled Dwarf Virus Isolates from Spain

**DOI:** 10.3390/plants13020250

**Published:** 2024-01-16

**Authors:** Ana Alfaro-Fernández, Rafael Taengua, Isabel Font-San-Ambrosio, Esmeralda Sanahuja-Edo, Rosa Peiró, Luis Galipienso, Luis Rubio

**Affiliations:** 1 Instituto Agroforestal Mediterráneo (IAM), Universitat Politècnica de València (UPV), 46022 Valencia, Valencia, Spain; analfer1@etsia.upv.es (A.A.-F.); mafonsa@upvnet.upv.es (I.F.-S.-A.); essaed@etsia.upv.es (E.S.-E.); 2 Instituto Valenciano de Investigaciones Agrarias (IVIA), 46113 Moncada, Valencia, Spain; taengua.rafael@gmail.com (R.T.); galipienso_lui@gva.es (L.G.); 3 Instituto de Conservación y Mejora de la Agrodiversidad Valenciana (COMAV), Universitat Politècnica de València (UPV), 46022 Valencia, Valencia, Spain; ropeibar@btc.upv.es

**Keywords:** negative-strand RNA virus, *Alphanucleorhabdovirus melongenae*, phylogeny, recombination, selection, gene flow, nucleotide diversity

## Abstract

The genetic variation and population structure of gene N (nucleocapsid) and part of gene L (replicase) from 13 eggplant mottle dwarf virus (EMDV) isolates from Spain were evaluated and compared with sequences of EMDV isolates from other countries retrieved from GenBank. Phylogenetic inference of part of gene L showed three main clades, one containing an EMDV isolate from Australia and the other two containing isolates from Iran and Europe, as well as four subclades. EMDV isolates from Spain were genetically very similar and grouped in a subclade together with one isolate from Germany and one from the UK. No new recombination events were detected in addition to one recombination previously reported, suggesting that recombination is rare for EMDV. The comparison of synonymous and non-synonymous rates showed that negative selection played an important role, and only two codons were under positive selection. Genetic differentiation (F_st_ test), phylogenetic and nucleotide diversity analyses suggest a unique introduction of EMDV to Spain and low gene flow with other countries. In contrast, Greece and Italy showed diverse populations with high gene flow between both.

## 1. Introduction

Understanding the genetic variability of virus populations and the factors involved in their evolution is crucial to developing accurate detection and diagnostic tools, implementing efficient disease control strategies and gaining insight into virus epidemiology [1]. The main evolutionary mechanisms shaping the genetic structure and variability of virus populations are mutation, recombination, selection, genetic drift and gene flow or migration [2,3]. RNA viruses have great potential for rapid evolution due to their rapid replication and high mutation rates since RNA replicases lack proofreading activity. Recombination is another source of genetic variation and the emergence of new plant viruses [4]. The genetic variation produced by mutation and recombination is limited by the interplay of selection, genetic drift and gene flow. Natural selection in plant viruses results from the competition among genetic variants differing in some aspects of the life cycle, such as replication, movement between plant cells and transmission to other plants by vectors, so those variants with more reproductive success (fitness) will pass to the next generation. Genetic drift is the change in the frequency of genetic variants in small populations by random chance. Viruses can undergo population bottlenecks or founder events in different life cycle steps such as cell-to-cell movement and transmission by vectors. Gene flow or migration of genetic variants favors genetic uniformity, whereas restricted migration leads to genetic differentiation between populations via selection and genetic drift.

Eggplant mottled dwarf virus (EMDV) has been assigned to the species *Alphanucleorhabdovirus melongenae* in the genus *Alphanucleorhabdovirus*, family *Rhabdoviridae*, order *Mononegavirales* [5]. The EMDV genome is a single-stranded, negative-sense RNA encapsidated by the nucleocapsid protein (N) and wrapped by a phospholipid membrane forming bacilliform particles. The EMDV genome contains seven open reading frames: N (nucleocapsid), X (unknown function), P (phosphoprotein, a polymerase cofactor), Y (putative movement protein), M (matrix protein, which connects the envelope to the ribonucleocapsid core), G (glycoprotein, which protrudes from the lipid envelope exterior) and L (RNA-dependent RNA polymerase) [5,6].

EMDV has a wide host range and infects important crops (e.g., eggplant, tomato, potato, pepper, cucumber and tobacco), ornamental (e.g., pittosporum, honeysuckle, pelargonium and hibiscus) and wild plants [7]. EMDV is transmitted by the leafhoppers *Agallia vorobjevi*, *Anaceratogallia laevis* and *A. ribauti* [8,9]. It is widespread in the Mediterranean basin and has been detected in Europe: Albania, Azerbaijan, Bulgaria, Croatia, France, Germany, Greece, Italy, Portugal, Slovenia, Spain, Türkiye and the UK; Asia: Afghanistan, Iran, Israel and Jordan; Africa: Algeria, Libya, Morocco and Tunisia; and Oceania: plants imported from Australia in New Zealand [7,10].

The genetic variability of EMDV and some evolutionary factors have been studied by sequence analyses of genes N, X, P, Y, M and G from seven isolates from Greece and one from Cyprus; part of gene L of these isolates from Greece and Cyprus, as well as eight from Italy and five from Spain [11,12]; and the complete sequence of one isolate from Iran [13]. In this work, the nucleotide sequence of gene N and part of gene L in 13 EMDV isolates from four regions of Spain and six hosts were determined and analyzed together with part of the gene L of another 5 Spanish isolates retrieved from GenBank. This work revealed that the population of EMDV in Spain was very homogeneous and had a low migration rate in contrast to those in Italy and Greece. A more comprehensive picture of the genetic variability and evolutionary mechanisms of EMDV globally was obtained by analyzing the entire genome of nine isolates—three from Italy, two from Greece and one each from Iran, Slovenia, Germany and the UK—as well as the N gene from six isolates from Greece and one from Cyprus; part of the L gene of six isolates from Greece, one from Cyprus, two from France and one from Australia; and a Y gene portion from one isolate from Azerbaijan.

## 2. Results

### 2.1. Sequencing EMDV Isolates from Spain

We analyzed the N gene (encoding the nucleocapsid) and a portion of the L gene (encoding the RNA-dependent RNA polymerase) to evaluate the genetic variability in EMDV in Spain and other countries. These genomic regions were chosen for three reasons: (i) they are frequently used to study plant virus genetic variability, which facilitates comparison between EMDV and other viruses; (ii) most EMDV sequences in GenBank either corresponded to or contained the selected gene L region, allowing for comparisons of variability in Spain and other countries; and (iii) because both genes, N and L, are separated in the genome, making them useful for recombination analysis.

The complete gene N and a region of gene L (named L1) of 13 EMDV isolates collected in several regions of Spain were amplified by RT-PCR, sequenced and compared with nucleotide sequences of other EMDV isolates retrieved from GenBank (Table 1). The nucleotide sequences were deposited in GenBank under accession numbers OR631742–OR631767.

EMDV isolates PV-1127 from Germany and 02923HTS from the UK showed the highest nucleotide identities with the Spanish isolates, ranging from 97.8 to 99.3% for gene N and from 97.3 to 99.1% for the genomic region L1 (Supplementary Tables S1 and S2). Comparison of the L1 region of the 13 Spanish isolates sequenced here and that of five Spanish isolates retrieved from GenBank showed that isolates S4 and S5 from the Granada province of Spain were similar to isolate 443/17, also collected in Granada (nucleotide identity > 99.0%). Isolates S1, S2 and S3 from the Spanish province of Malaga showed lower nucleotide identity with the other Spanish isolates (ranging from 96.3 to 97.1%). Isolate 203/09 also differed from the other Spanish isolates (nucleotide identity, 96.9–97.4%). The rest of the Spanish isolates showed more similar sequences (nucleotide identity 98.1–99.5%).

### 2.2. Phylogenetic Relationships among Worldwide EMDV Isolates

**Figure 1:**
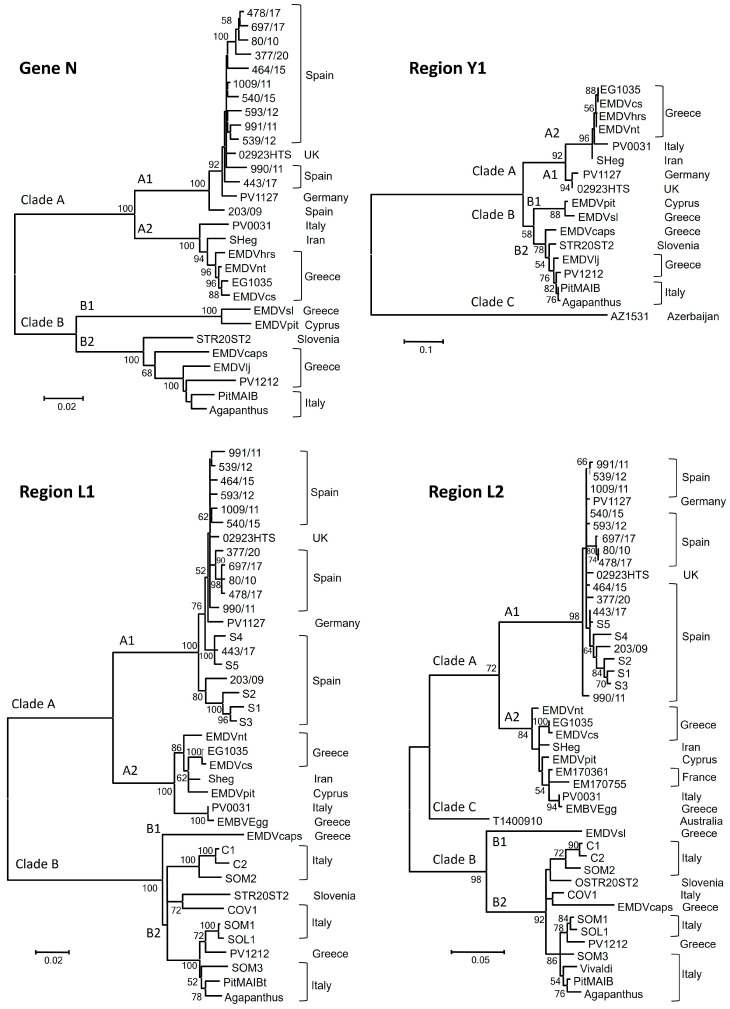
Unrooted maximum likelihood phylogenetic trees of four EMDV genomic regions: gene N, Y1 (gene Y, nucleotide position 277–865), L1 (gene L, position 1090–1965) and L2 (gene L, position 1375–1670) of EMDV isolates from different countries (see Table 1). Horizontal bars are proportional to evolutionary distances, and bootstrap values greater than 50% are in the nodes. Clades A, B and C and subclades A1, A2, B1 and B2 are indicated in the corresponding branches.

Phylogenetic analysis of the N gene of 29 EMDV isolates from eight countries showed two main clades, A and B, each divided into two subclades, A1, A2, B1 and B2 (Figure 1). Subclade A1 was composed of all isolates from Spain, one isolate from Germany and another from the UK, forming a homogenous and undifferentiated group (the minimum nucleotide identity between isolate pairs was 97.3%). Subclade A2 was composed of isolates from Iran, Italy and Greece. The minimum nucleotide identity within subclade A2 was 97.1%, whereas nucleotide identity between subclades A1 and A2 varied from 91.7 to 92.9%. Subclade B1 had two isolates, one from Greece and another from Cyprus (nucleotide identity of 97.2%), and B2 was composed of isolates from Italy, Greece and Slovenia. The minimum nucleotide identity within subclade B2 was 94.1%, and nucleotide identities between subclades B1 and B2 ranged from 89.0 to 89.6%. Nucleotide identity between clades A and B ranged from 85.3 to 87.6%.

Phylogenetic trees of the L1 genomic region (876 nt) of 39 isolates from eight countries and the L2 region (296) of 44 isolates from 10 countries (Figure 1) had the same topology with high values of bootstrap for the external nodes, but L1 showed better resolution in the external nodes. The phylogenetic tree of L2 comprised three main clades: A, B and C. Clades A and B and their divisions (A1, A2, B1 and B2) contained isolates in the same clades and subclades of the phylogenetic tree of gene N. Clade C contained the EMDV isolate T14_00910 from Australia. Nucleotide identities were 81.4–86.4% between clades A and B, 84.5–88.9% between A and C and 84.5–87.4% between B and C. Subclade A1 contained 1 isolate from Germany, 1 from the UK, the 13 Spanish isolates sequenced here and another 5 Spanish isolates retrieved from GenBank. Subclade A2 contained isolates from Iran, Greece, Italy and France and the isolate EMDVpit from Cyprus that was in the subclade B1 for gene N. Clades A1 and A2 had minimum nucleotide identities of 96.3% and 95.6%, respectively, and the nucleotide identity between these two clades ranged from 87.5 to 91.2%. Subclade B1 had only isolate, EMDVsl from Greece, and subclade B2 contained isolates from Slovenia, Italy and Greece. The minimum nucleotide identity within clade B2 was 90.5%, and that between B1 and B2 ranged from 85.8 to 88.5%.

The phylogenetic tree of a 588 nt region of gene Y (named Y1) in 17 EMDV isolates from eight countries showed three main clades: A, B and C (Figure 1). Clades A and B and the subdivisions A1, A2, B1 and B2 contained the same isolates as the equivalent clades for the N gene and the L1 and L2 genomic regions. Nucleotide identities within each subclade were higher than 94.0%, 91.3–92.9% between subclades A1 and A2 and 88.6–90.1% between B1 and B2. Clade C contained the isolate AZ15-31 from Azerbaijan, showing high divergence from the other isolates (nucleotide identities between 74.4 and 76.4%, whereas clade A and B had nucleotide identities ranging from 83.3 to 88.8%. Blastn analysis showed that isolate AZ15-31 had nucleotide identities of 82.6% with tomato alphanucleorhabdovirus 1 (TARV1) and 78.0% with physostegia chlorotic mottle virus (PhCMoV), both being distinct species belonging to the genus *Alphanucleorhabdovirus*.

The phylogenic trees showed no correlation between genetic distances and hosts. For example, for gene N, the isolate EMDVcs from cucumber was genetically close to the isolate EG1035 from eggplant and genetically distant to another cucumber isolate, PV1212. Another example is for the genomic region L2, where the isolate EM170361 from tomato was close to the eggplant isolate EG1035 and the cucumber isolate EMDVcs but distant from another tomato isolate, Sol1, which was close to the cucumber isolate PV1212.

### 2.3. Recombination between EMDV Variants

Several sets of nucleotide sequences were analyzed with seven recombination detection methods implemented in the package RDP4 [23]. Analysis of the complete genome (13163 nt) or each gene individually (N, X, P, Y, M, G and L) of the nine EMDV isolates with the genome completely sequenced (isolates SH-eg, STR20ST2, PV-1127, 02923HTS, Pit-MAIB, Agapanthus, PV-0031, EG1035 and PV-1212, see Table 1) showed no recombination with any of these methods. Also, no recombination was found after analyzing the N gene of 29 EMDV isolates or two regions of gene L: L1 (876 nt) of 39 EMDV isolates and L2 (296 nt) of 44 EMDV isolates.

Finally, the sequences of N and L1 were concatenated for each isolate separately for the 13 Spanish isolates sequenced here, and isolates EMDVcs, EMDVnt and EMDVpit, along with equivalent sequences which were prepared from the nine isolates with the complete genome sequenced. Thus, we generated sequences composed of gene N and the region L1 from 25 EMDV isolates to use as input. The seven methods performed using the RDP4 package detected one recombination event for isolate EMDVpit involving an ancestor of isolate STR20ST2 as the major parental and an SH-eg ancestor as the minor parental. The phylogenetic trees confirmed this recombination event since EMDVpit was placed in different clades depending on the genomic region analyzed. Thus, isolates EMDVpit and STR20ST2 are in clade B and SH-eg is in clade A in the phylogenetic trees of genes N and Y, while in the phylogenetic tree of gene L, EMDVpit and SH-eg are in clade A and STR20ST2 is in clade B (Figure 1). The nucleotide identity of EMDVpit was higher with SH-eg (98.2%) than with STR20ST2 (85.0%) for gene L, whereas the opposite occurred for genes N and Y (Table 2). This result suggests that the breaking point could exist between the genes Y and L. This recombination even was detected previously in the gene G, but only the minor parental was found [11].

### 2.4. Genetic Variation, Selection and Coevolution in Different EMDV Genes

Analysis of the nine completely sequenced EMDV isolates with the genome showed differences in genetic variability between genes. Nucleotide diversity was similar for genes N, P, M and G and slightly higher for genes X and Y (Table 3). Surprisingly, gene Y had the highest nucleotide diversity and the lowest proportion of segregating sites. The ratio between non-synonymous and synonymous rates (dN/dS) was less than one for every gene, indicating negative selection, ranging from 0.010 for gene Y to 0.123 for gene X. Evaluation of selection at individual codons (amino acids) showed that around 10% of the amino acids were under negative (purifying) selection, and amino acid positions 449 of gene N and 10 of gene X were under positive (diversifying) selection.

Finally, we analyzed coevolution among different amino acid sites within each EMDV protein for EMDV isolates with the complete genome sequenced. Only positions 190 and 376 of N and 57 and 86 of X were coevolving. However, analysis of gene N from 29 EMDV isolates showed six coevolving amino acid pairs: 68–157, 169–193, 218–423, 228–442, 240–432 and 381–399. Analysis of L1 showed ten coevolving amino acid pairs: 377–541, 393–402, 406–413, 417–614, 422–624, 441–535, 519–541, 521–589, 568–651 and 628–654 (position relative to L1).

### 2.5. Genetic Differentiation of EMDV Populations

Genetic differentiation between EMDV populations, determined by the gene flow (migration) rate, was estimated by calculating the nucleotide diversity within and between geographical regions and using the statistic F_st_.

The nucleotide diversity of EMDV within Italy or Greece was high (>0.100) and about ten times lower than that within Spain (Table 4). Comparison between countries showed nucleotide diversities in the same order as those within Italy or Greece. F_st_ values suggest high gene flow between Italy and Greece and low gene flow between both countries and Spain.

To study the population structure of EMDV in Spain, four regions were considered: (i) Malaga province, (ii) Granada province, (iii) Almeria province and (iv) the rest, composed of Navarra, Pontevedra, Valencia and Zaragoza provinces. Nucleotide diversities within and between regions were lower than 0.020, except between Malaga and the other Spanish regions, with values around 0.040 (Table 5). F_st_ values showed two genetically differentiated populations of EMDV in Spain with very low gene flow between them. One was in Malaga province, and the other corresponded to the other provinces of Spain, which showed high gene flow between them.

## 3. Discussion

EMDV genetic variability was relatively high compared to that of most plant viruses [24,25], which should be considered for detection and disease control [1,26]. Thus, we had to design primers based on conserved sequence stretches for the gene L region since RT-PCR with other primers [12] failed with some Spanish EMDV isolates.

Phylogenetic analyses of genomic regions N, Y1, L1 and L2 were congruent among themselves and with those from previous studies [11,12,21]. These phylogenetic trees show a certain geographic structure since the Australian isolate is in clade C, all Spanish isolates are in subclade A1 and most Italian isolates are in subclade B2. However, Greece contains EMDV isolates in clades A (subclade 2) and B (B1 and B2), and France contains isolates in subclade A2, which are more similar to distant isolates like SH-eg from Iran than isolates located in Spain, Germany and the UK. More isolates from different countries should be analyzed to gain insight into the migration routes of EMDV [27].

Phylogenetic analysis of the Y1 genomic region showed that isolate AZ15-31 from Azerbaijan [22] formed a different clade and was divergent from the other EMDV isolates, with a nucleotide identity of about 75.0%, which is in the same range as EMDV with some other species of the genus *Alphanucleorhabdovirus*. One of the three criteria to differentiate species within the genus *Alphanucleorhabdovirus* is that the nucleotide sequence identity of entire genomes must be less than 75.0% [5]. Thus, the entire genome of isolate AZ15-31 should be sequenced to assign it to EMDV or another species of the genus *Alphanucleorhabdovirus* such as *Alphanucleorhabdovirus physostegiae* (physostegia chlorotic mottle virus, PhCMoV) or to propose a new species in this genus.

Recombination seems to be uncommon for EMDV since only one recombination event was detected [11]. Homologous recombination is rare in negative-strand RNA viruses [28,29] in contrast to positive-strand RNA viruses [4,30,31]. Recombination events found in negative-strand RNA viruses might be artifacts from mixed infections or laboratory contamination [31]. So, the presence of recombinant variants should be confirmed by analyzing within-plant viral populations [32].

Some EMDV isolates collected in distant places and times showed similar nucleotide sequences. For example, EMDV isolates 1009/11 collected in Spain in 2011 and 02923HTS collected in the UK in 2022 showed 99.3% nucleotide identity. This suggests genetic stability by strong negative selection within a narrow adaptative peak [33] for the phylogenetic subclade A1. Genetic stability has been found in other plant RNA viruses [34,35]. A comparison of synonymous and non-synonymous substitution rates showed that most EMDV genes were subjected to strong negative selection due to functional restrictions. This is frequent for plant viruses [24,36,37] since proteins can have multiple domains and functions in the virus life cycle [38,39]. Only two codons were under diversifying positive selection, which could be result of adaptation to a new host, vector or environment [40]. Positive selection has been detected in other plant viruses, for example, in the substitution of one amino acid (codon) in the movement protein of tomato spotted wilt virus (TSWV), which is an adaptation to overcome resistance in tomato [41].

The low genetic diversity and the low genetic flow of EMDV isolates from Spain with respect to those from Greece and Italy suggest a unique introduction of EMDV to Spain. The EMDV population within Spain was genetically very homogeneous with high gene flow, except in Malaga province, which showed slight divergence and low gene flow with the rest of Spain. EMDV might have undergone genetic drift after a founder or bottleneck event or experienced adaptation to new conditions in Malaga. Further analysis of additional Malaga EMDV isolates from various hosts would be necessary to verify whether this slight divergence is present in other isolates.

In contrast, Greece and Italy had diverse EMDV populations with high gene flow between both. Greece showed the highest diversity with EMDV isolates in clades A and B, so the center of diversification and dispersion might be in Greece or nearby. Some plant viruses could have dispersed and diversified from Middle East [30], where farming originated. More EMDV sequences from these countries and from Middle East would be necessary to test this hypothesis.

## 4. Material and Methods

### 4.1. Virus Isolates

We collected 658 plants from different hosts and provinces of Spain from 2009 to 2020 and analyzed them by DAS-ELISA for EMDV (Loewe, Sauerlach, Germany). Only 13 plants were positive for EMDV (Table 1) and were used for RT-PCR, sequencing and sequence analyses.

### 4.2. Primer Design

Primers were designed with Oligo 7 software [42] from conserved stretches of the nine EMDV isolates with complete genome sequences available (GenBank accessions KC905081, OL472111, OQ847408, OQ716555, LN680656, KJ082087, MW854257, FR751552 and OL584369) to minimize false negatives [1]. Two primer pairs (N1F2/N2bR and N2F2/N1R2) comprising two overlapping regions encompassing gene N and primers PL1a/Pl2B covering a 1053 nt region of gene L were designed (Table 6). An 876 nt region of the PL1a/Pl2B PCR product was selected for nucleotide sequence analysis and was named L1.

### 4.3. Nucleic Acid Purification, RT-PCR and Sequencing

Total RNAs were extracted from 100 mg of leaf tissues using the silica-capture protocol [43] and stored at −20 °C until use.

RT was performed by denaturing 3 µL of total RNA extracts by heating at 95 °C for 5 min, incubating them on ice for 1 min and adding them to a 10 µL reaction mixture containing 5 mM DTT, 1 mM of each dNTP, first-strand buffer, 0.4 µM of primer EMDV-N1F2 (Table 6), 10 units of Ribolock RNAse Inhibitor (Thermo Fisher Scientific, Waltham, MA, USA) and 40 units of SuperScript IV Reverse Transcriptase (Thermo Fisher Scientific, Waltham, MA, USA). The reaction mixture was incubated at 50 °C for 10 min and at 80 °C for 10 min to inactivate the reaction. Then, 1 µL of the RT product was added to a 20 µL reaction mixture containing CloneAmp HiFi PCR Premix (Takara, Kusatsu, Shiga, Japan) and 0.2 µM of primers. A Mastercycler epgradient thermal cycler (Eppendorf, Hamburg, Germany)was used with the following thermocycling conditions: denaturation at 98 °C for 1 min; 35 cycles of 98 °C for 10 s, 55 °C for 10 s and 72 °C for 8 s; and an extension step at 72 °C for 1 min. PCR products were analyzed by electrophoresis in 2% agarose gels and visualized with Gel Red (Biotium Inc., Fremont, CA, USA) under UV light. The PCR products were purified using the mi-PCR purification kit (Metabion, Planegg, Germany) and the DNA concentration was estimated with a NanoDrop™ 1000 spectrophotometer (Thermo Fisher Scientific, Waltham, MA, USA). Sanger sequencing of the purified RT-PCR products was carried out on an ABI 3730XL capillary sequencer (Applied Biosystems, Waltham, MA, USA) by Eurofins Genomic GATC service (Ebersberg, Germany).

### 4.4. In Silico Analyses of Nucleotide Sequences

Nucleotide sequences were aligned with the algorithm CLUSTALW [44] implemented in MEGA X software ver. 10.0.5 [45].

Nucleotide identities were estimated from the formula 100 × (1 − p-distance) with MEGA X. Nucleotide diversity was calculated as the mean distance between sequence pairs using the best fitting substitution model based on the lowest Bayesian information criterion (BIC) scores estimated with MEGA X. The T92 + G model was used since it was the first or second model with the lowest BIC for the distinct genomic regions analyzed and was available in further analyses.

Unrooted phylogenetic trees were inferred from the nucleotide sequences with the maximum likelihood method [46], implemented in MEGA X, under the T92 + G substitution model and five discrete gamma rates. The statistical significance of nodes was estimated with 500 bootstrap pseudoreplicates [47].

Possible recombination events between EMDV isolates were analyzed with the algorithms GENECONV, BOOTSCAN, MAXCHI, SISCAN, 3SEQ, LARD and RDP implemented in the RDP4 package [23]. Only recombination events detected by at least five different methods were considered.

Natural selection at the amino acid level was estimated with MEGA X by separately analyzing the rate of non-synonymous (dN) and synonymous (dS) substitutions with the Pamilo–Bianchi–Li method [48]. The rate between dN and dS provides information on the sign and intensity of selection. Selection across the genomic coding regions was studied by estimation of the rates of dN and dS at each codon using the fixed effects likelihood method [49] implemented in the adaptive evolution server Datamonkey 2.0 [50]. Coevolving amino acid sites within each EMDV protein were identified with Spidermonkey-BGM [51] implemented in Datamonkey.

The level of genetic differentiation between populations, determined by gene flow (migration), was estimated with the statistic F_st_ [52] implemented in the program DNASP v6.12 [53]. F_st_ values vary between zero, corresponding to genetically undifferentiated populations, and one, indicating genetically isolated populations. An absolute value of F_st_ > 0.333 suggests infrequent gene flow.

## Figures and Tables

**Table 1:** Eggplant mottled dwarf virus (EMDV) isolates analyzed in this study.

Isolate	Country	Region	Year	Host	Genes ^1^	GenBank
203/09	Spain	Valencia	2009	*Pittosporum tobira*	N, L1	OR631742, OR631755
80/10	Spain	Almería	2010	*Solanum melongena*	N, L1	OR631743, OR631756
990/11	Spain	Almería	2011	*Solanum lycopersicum*	N, L1	OR631744, OR631757
991/11	Spain	Almería	2011	*Cucumis sativus*	N, L1	OR631745, OR631758
539/12	Spain	Granada	2012	*Solanum lycopersicum*	N, L1	OR631746, OR631759
443/17	Spain	Granada	2017	*Solanum melongena*	N, L1	OR631747, OR631760
478/17	Spain	Granada	2017	*Solanum melongena*	N, L1	OR631748, OR631761
697/17	Spain	Granada	2017	*Solanum melongena*	N, L1	OR631749, OR631752
377/20	Spain	Granada	2020	*Solanum melongena*	N, L1	OR631750, OR631763
593/12	Spain	Navarra	2012	*Capsicum annuum*	N, L1	OR631751, OR631764
1009/11	Spain	Pontevedra	2011	*Capsicum annuum*	N, L1	OR631752, OR631765
464/15	Spain	Valencia	2015	*Podranea ricasoliana*	N, L1	OR631753, OR631766
540/15	Spain	Zaragoza	2015	*Pittosporum tobira*	N, L1	OR631754, OR631767
S1	Spain	Malaga	2011	*Hibiscus rosa-sinensis*	L1	HG916821 [12]
S2	Spain	Malaga	2011	*Hibiscus rosa-sinensis*	L1	HG916822 [12]
S3	Spain	Malaga	2011	*Hibiscus rosa-sinensis*	L1	HG916823 [12]
S4	Spain	Granada	2011	*Hibiscus rosa-sinensis*	L1	HG916824 [12]
S5	Spain	Almeria	2013	*Cucumis sativus*	L1	HG916825 [12]
SH-eg	Iran		2011	*Solanum melongena*	N, X, P, Y, M, G, L	KC905081 [13]
STR20ST2	Slovenia		2020	*Solanum lycopersicum*	N, X, P, Y, M, G, L	OL472111 [14]
PV-1127	Germany		2014	*Hydrangea macrophylla*	N, X, P, Y, M, G, L	OQ847408 [15]
02923HTS	UK		2022	*Pittosporum tobira*	N, X, P, Y, M, G, L	OQ716555 [16]
Pit-MAIB	Italy		2008	*Pittosporum tobira*	N, X, P, Y, M, G, L	LN680656 [17]
Agapanthus	Italy		2012	*Agapanthus sp.*	N, X, P, Y, M, G, L	KJ082087 [18]
PV-0031	Italy		1987	*Solanum melongena*	N, X, P, Y, M, G, L	MW854257
EG1035	Greece	Thessaloniki	2009	*Solanum melongena*	N, X, P, Y, M, G, L	FR751552 [6]
PV-1212	Greece	Thessaloniki	2017	*Cucumis sativus*	N, X, P, Y, M, G, L	OL584369
EMDVcs	Greece	Katerini	2008	*Cucumis sativus*	N, L1	HG794532, HG794539 [11,19]
EMDVnt	Greece	Thessaloniki	2006	*Nicotiana tabacum*	N, L1	HG794534, HG794540 [11,19]
EMDVsl	Greece	Thessaloniki	2010	*Solanum lycopersicum*	N, L1	HG794535, HG794543 [11,19]
EMDVpit	Cyprus	Nicosia	2011	*Pittosporum tobira*	N, L1	HG794531, HG794544 [11,19]
EMDV-Egg	Greece		2007	*Hibiscus rosa-sinensis*	L1	AM922322 [19]
EMDVhrs	Greece	Thessaloniki	2009	*Lonicera japonica*	N	HG794541 [11]
EMDVlj	Greece	Sifnos island	2010	*Capparis spinosa*	N	HG794542 [11]
EMDV-caps	Greece	Santorini	2005	*Capparis spinosa*	N	HG794545 [11]
EMDV-capsL	Greece	Rhodes	2009	*Solanum melongena*	L1	HG794533 [19]
EMDVsm	Greece	Thessaloniki	2010	*Hibiscus rosa-sinensis*	L1	HG794536 [19]
Vivaldi	Italy	Emilia-Romagna	2012	*Solanum tuberosum*	L1	KC760150 [20]
C1	Italy	Calabria	2010	*Hibiscus rosa-sinensis*	L1	HG916814 [12]
C2	Italy	Calabria	2010	*Hibiscus rosa-sinensis*	L1	HG916815 [12]
SOM-1	Italy	Campania	2011	*Solanum melongena*	L1	HG916816 [12]
SOM-2	Italy	Campania	2011	*Solanum melongena*	L1	HG916817 [12]
SOM-3	Italy	Campania	2013	*Solanum melongena*	L1	HG916818 [12]
SOL-1	Italy	Campania	2010	*Solanum lycopersicum*	L1	HG916819 [12]
COV-1	Italy	Emilia-Romagna	1990	*Codiaeum variegatum*	L1	HG916820 [12]
EM170361	France		2017	*Solanum lycopersicum*	L2	MN990976 [10]
EM170755	France		2017	*Cucumis sativus*	L2	MN990977 [10]
T14_00910	Australia		2014	*Hibiscus syriacus*	L2	KJ742827 [21]
AZ15-31	Azerbaijan		2015	*Cucumis sativus*	Y1	MG964325 [22]

^1^ L1 = position 1090–1965 of gene L, L2 = position 1375–1670 of gene L, Y1 = position 277–865 of gene Y.

**Table 2:** Nucleotide identity between the recombinant isolate EMDVpit and the parental isolates SH-eg and STR20ST2.

Isolates	N	Y	L
SH-eg/STR20ST2	87.6	86.7	85.2
SH-eg/EMDVpit	85.9	84.4	98.2
STR20ST2/EMDVpit	89.2	90.1	85.0

**Table 3:** Population genetics parameters of different coding regions of EMDV.

Gene	S	π	dN	dS	dN/dS	N	NSe	Pos	Neg
N	0.209	0.167	0.012	0.443	0.027	1431	299	1 (449)	140
X	0.320	0.225	0.072	0.587	0.123	294	94	1 (10)	24
P	0.226	0.170	0.021	0.437	0.048	885	200	0	86
Y	0.189	0.241	0.005	0.485	0.010	864	163	0	112
M	0.228	0.178	0.017	0.474	0.036	756	172	0	96
G	0.223	0.163	0.012	0.501	0.023	1848	412	0	231
L	0.224	0.179	0.013	0.495	0.028	5841	1309	0	761

S = proportion of segregating (polymorphic) sites. π = nucleotide diversity (mean nucleotide differences per site between sequence pairs, dN = average number of non-synonymous substitutions per non-synonymous site. dS = average number of synonymous substitutions per synonymous site. N = number of nucleotide sites of each gene. NSe = number of segregating sites. Pos = number of codons under positive selection (between parentheses are the codon positions). Neg = number of codons under negatives selection.

**Table 4:** Genetic diversity and differentiation of EMDV populations in three Mediterranean countries.

Gene	N		Italy	Greece	Spain
N	3	Italy	**0.166** **±** **0.015**		
	8	Greece	0.148 ± 0.012 (−0.068)	**0.177** **± 0.014**	
	13	Spain	0.187 ± 0.015 (0.524)	0.203 ± 0.017 (0.487)	**0.019** **± 0.002**
L1	10	Italy	**0.110** **± 0.013**		
	6	Greece	0.239 ± 0.036 (0.277)	**0.206** **± 0.031**	
	18	Spain	0.285 ± 0.046 (0.688)	0.206 ± 0.026 (0.475)	**0.025** **± 0.003**

Gene = Genomic regions analyzed: gene N (1431 nt) and L1, a segment of gene L (876 nt). N = number of isolates per country and genomic region. N values < 5 are too low to represent a country and should be interpreted with caution. Nucleotide diversity estimated as mean nucleotide distance between sequence pairs using the substitution model T92 + G, where G = 0.27. Nucleotide diversity values within countries are in bold on the diagonal, and diversity values between countries are below the diagonal. F_st_ values are between parentheses.

**Table 5:** Genetic diversity and differentiation of EMDV populations in Spain.

N		Malaga	Granada	Almeria	Rest
3	Malaga	**0.018** **±** **0.003**			
6	Granada	0.039 ± 0.005 (0.520)	**0.016** **± 0.004**		
4	Almeria	0.040 ± 0.005 (0.492)	0.016 ± 0.002 (−0.094)	**0.019** **± 0.003**	
5	Rest	0.038 ± 0.005 (0.472)	0.019 ± 0.003 (0.046)	0.019 ± 0.003 (0.011)	**0.018** **± 0.004**

Genomic regions analyzed: L1, a segment of gene L (876 nt). N = number of isolates per each region of Spain: Malaga, Granada and Almeria provinces, and Rest corresponds to Navarra, Pontevedra, Valencia and Zaragoza provinces. Nucleotide diversity estimated as mean nucleotide distance between sequence pairs using the substitution model T92 + G, where G = 0.27. Nucleotide diversities within Spanish regions are in bold on the diagonal, and diversities between countries are below the diagonal. F_st_ values are between parentheses.

**Table 6:** Oligonucleotide primers designed for RT-PCR.

Primer	Sequence	Position ^1^	PCR Size ^2^
EMDV-N1F2	ATGACTATTTAAATAAAACCCAACAAC	181–207	914
EMDV-N2bR	AGTGGTGAGGAGCATCTTGTA′	1074–1094	
EMDV-N2F2	TTATTCAAGGCCTAGATACTTACACTG	922–948	846
EMDV-N1R2	CACACACCACAAACATAGACTAGATAC	1741–1767	
EMDV-PL1a	ATGGGGGCATCCTATCATAGA-	8165–8185	1053
EMDV-PL2b	GCGACGTACTTTATATCACACACTGTCAT	9189–9216	

^1^ Nucleotide position with respect to the sequence retrieved from GenBank with the accession number LN680656. ^2^ PCR amplicon size (bp).

## Data Availability

The 26 nucleotide sequences generated in this work were deposited in GenBank under accession numbers OR631742–OR631767, 3 October 2023.

## Supplementary Materials

The following supporting information can be downloaded at: https://www.mdpi.com/article/10.3390/plants13020250/s1. Supplementary Table S1. Nucleotide identity of the gene N between EMDV isolates from Spain and one isolate from Germany and the UK. Supplementary Table S2. Nucleotide identity of the genomic region L1 (position 1090–1965 of the gene L) between EMDV isolates from Spain and one isolate from Germany and the UK.
