# Supplementary material for: Genetic Variation and Evolutionary Analysis of Eggplant Mottled Dwarf Virus Isolates from Spain
Source: Plants (Basel). 2024 Jan 16;13(2):250. doi: 10.3390/plants13020250 (PMC10818716; doi:10.3390/plants13020250)
Supplement: Supplementary file 1 [file plants-13-00250-s001.zip › plants-2797716-supplementary.pdf]

Supplementary Table S1. Nucleotide identity of the gene N between EMDV isolates from Spain and one isolate from Germany and the UK.

Isolate	Origin														
80/10	Almeria														
990/11	Almeria	97.8													
991/11	Almeria	98.3	98.2												
539/12	Granada	98.3	98.3	99.1											
443/17	Granada	98.0	98.3	98.4	98.5										
478/17	Granada	99.2	98.0	98.5	98.5	98.3									
697/17	Granada	99.0	97.8	98.3	98.3	98.0	99.4								
377/20	Granada	98.6	97.7	98.1	98.1	98.0	98.8	98.9							
593/12	Navarra	98.1	98.2	98.6	98.7	98.4	98.3	98.1	98.0						
1009/11	Pontevedra	98.7	98.7	99.0	99.2	99.0	98.9	98.7	98.5	99.0					
203/09	Valencia	97.4	97.3	97.6	97.8	97.8	97.5	97.3	97.1	97.8	98.2				
464/15	Valencia	97.9	97.9	98.1	98.3	98.0	98.1	97.9	97.8	98.1	98.7	97.3			
540/15	Zaragoza	98.4	98.3	98.6	98.7	98.5	98.6	98.4	98.3	98.7	99.3	98.0	98.3		
PV1127	Germany	98.0	98.0	98.5	98.5	98.4	98.2	98.0	97.8	98.5	98.9	98.0	98.0	98.6	
02923HTS	UK	98.4	98.5	98.7	98.9	98.7	98.6	98.5	98.3	98.7	99.3	98.2	98.4	98.9	98.6

Supplementary Table S2. Nucleotide identity of the genomic region L1 (position 1090-1965 of the gene L) between EMDV isolates from Spain and one isolate from Germany and the UK.

Isolate	Origin																			
80/10	Almeria																			
990/11	Almeria	98.5																		
991/11	Almeria	98.2	98.5																	
S5	Almeria	97.8	98.4	98.1																
539/12	Granada	98.7	99.1	99.0	98.4															
443/17	Granada	98.1	98.6	98.3	99.3	98.6														
478/17	Granada	99.5	98.5	98.2	97.8	98.7	98.1													
697/17	Granada	99.5	98.6	98.3	97.9	98.9	98.2	99.5												
377/20	Granada	99.0	98.6	98.3	97.9	98.9	98.2	99.0	99.1											
S4	Granada	97.6	98.2	97.8	98.9	98.2	99.1	97.6	97.7	97.7										
593/12	Navarra	98.6	99.0	98.6	98.3	99.2	98.5	98.6	98.7	98.7	98.1									
1009/11	Pontevedra	98.6	99.0	98.7	98.3	99.2	98.5	98.6	98.7	99.0	98.1	99.1								
203/09	Valencia	97.3	97.6	96.9	97.0	97.5	97.3	97.3	97.4	97.1	97.3	97.4	97.4							
464/15	Valencia	98.6	99.0	98.9	98.3	99.2	98.5	98.6	98.7	98.7	98.1	99.1	99.2	97.4						
540/15	Zaragoza	98.3	98.6	98.3	97.9	98.9	98.2	98.3	98.4	98.4	97.7	98.7	99.0	97.0	98.7					
S1	Malaga	96.5	96.9	96.2	96.3	96.8	96.6	96.5	96.6	96.6	96.3	96.7	96.7	96.9	96.7	96.3				
S2	Malaga	96.3	97.0	96.3	96.7	96.9	96.9	96.3	96.5	96.5	96.7	96.8	96.8	96.9	96.8	96.7	97.8			
S3	Malaga	96.7	97.1	96.5	96.8	97.0	97.0	96.7	96.8	96.8	96.8	96.9	96.9	97.4	96.9	96.6	98.9	98.3		
PV1127	Germany	98.1	98.4	98.1	97.9	98.6	98.2	98.1	98.2	98.2	97.7	98.5	98.5	97.3	98.5	98.2	96.1	96.2	96.3	
02923HTS	UK	98.6	98.9	98.5	98.2	99.1	98.4	98.6	98.7	98.6	97.9	99.0	99.0	97.4	99.0	98.6	96.6	96.7	96.8	98.4
